# Facial Artery Musculomucosal Flap in Alveolar Cleft Surgery

**DOI:** 10.22038/IJORL.2021.55381.2901

**Published:** 2021-11

**Authors:** Amin Rahpeyma, Saeedeh Khajehahmadi

**Affiliations:** 1 *Oral and Maxillofacial Diseases Research Center, Mashhad University of Medical Sciences, Mashhad, Iran* *.*; 2 *Department of Oral and Maxillofacial Pathology, School of Dentistry, Mashhad University of Medical Sciences, Mashhad, Iran.*

**Keywords:** Buccinator flap, Cleft palate, Palatal fistula

## Abstract

**Introduction:**

Large anterior palatal fistula and special alveolar clefts, such as edentulous atrophic premaxilla and absent premaxilla (premaxillectomy or agenesis), as well as wide unilateral alveolar cleft, are complicated cases in alveolar cleft bone grafting surgery. A superiorly-based buccinator myomucosal flap is suitable in this regard.

**Materials and Methods:**

The cleft patients whose large anterior palatal fistula and superiorly based buccinator myomucosal flap had been used for palatal or alveolar reconstruction were recruited in the study. The reconstruction method of the nasal floor, follow-up time, and hospital length of stay were recorded.

**Results:**

A total of 10 patients had been treated by this method. The majority of them were male (6/10), the age range of the patients was 14-25 years. All flaps survived and a case of partial necrosis occurred.

**Conclusion:**

As evidenced by the obtained results, a superiorly-based facial artery musculomucosal flap is suitable when the palatal fistula is continuous with the alveolar cleft. Transmaxillary transfer is the other option in patients with closed maxillary arch.

## Introduction

Small palatal fistula in patients with a cleft is the result of wound dehiscence of mobilized flaps used for primary palatoplasty (1). Moreover, large fistulas develop from repeated unsuccessful attempts or total flap necrosis from damage to nutrient blood vessels (2). Closure of these recalcitrant fistulas does not follow the common method of nasal side closure by hinge flaps and oral side coverage with local flaps. 

In this case, we should seek help from the tongue, temporal, temporoparietal, submental, and buccal flaps (3-7). Free flaps, such as radial forearm flap, are the last choice (8).

Special alveolar clefts, such as edentulous atrophic premaxilla and absent premaxilla (premaxillectomy or agenesis), as well as wide unilateral alveolar cleft plus large oronasal fistula in the anterior palate, are other complicated examples in alveolar cleft bone grafting surgery. 

Superiorly-based buccinator myomucosal flap is an excellent method. The details of the surgery and the validity of this technique are explained.

## Materials and Methods

All procedures were approved by the Institutional Ethics Committee (IR.MUMS. REC.1393.46). The cleft patients whose large anterior palatal fistula and superiorly-based buccinator myomucosal flap had been used for palatal or alveolar reconstruction were included. 

The reconstruction method of the nasal floor, follow-up time, and hospital length of stay were recorded. 

A superiorly-based facial artery myomucosal (FAMM) flap was used to cover the outer side of the bone graft. This flap outlined anterior to the Stensen duct at an oblique direction posteriorly toward the mandibular angle.

**Fig 1:**
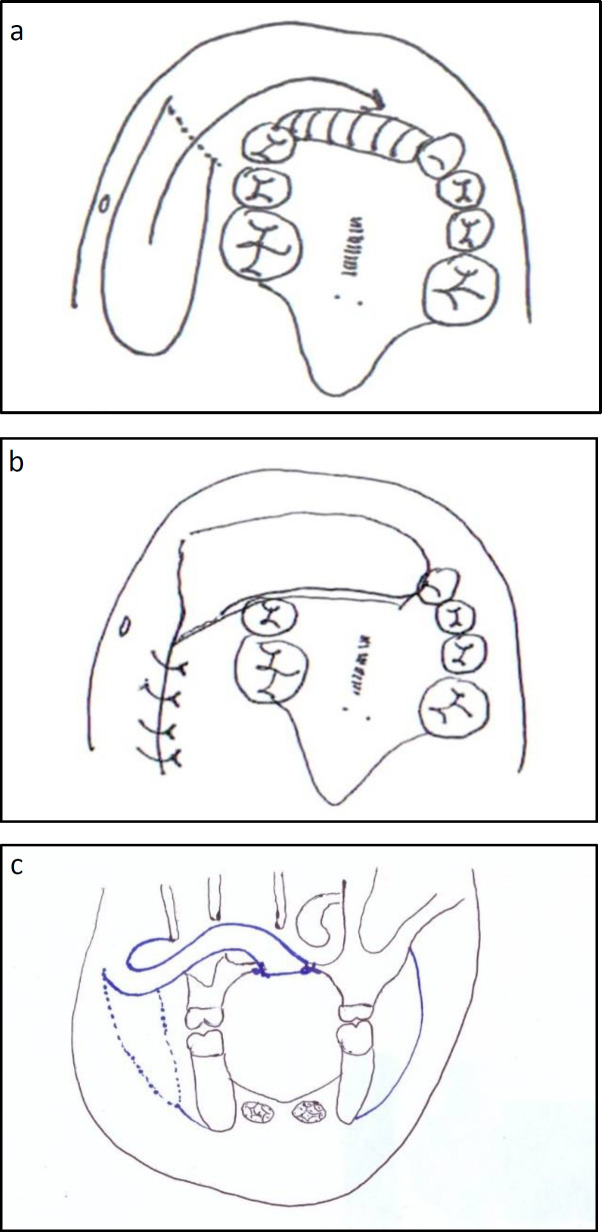
Schematic picture of superiorly based FAMM flap .a: Design of flap anterior to Stensen duct opening. b) Coverage of anterior maxillary bone graft. c) Transmaxillary transfer

 The width of the free end of the flap was considered equal to the defect, and the flap length was the same as the distance between the upper buccal vestibule and the recipient site (Figure 1). 

**Fig 2:**
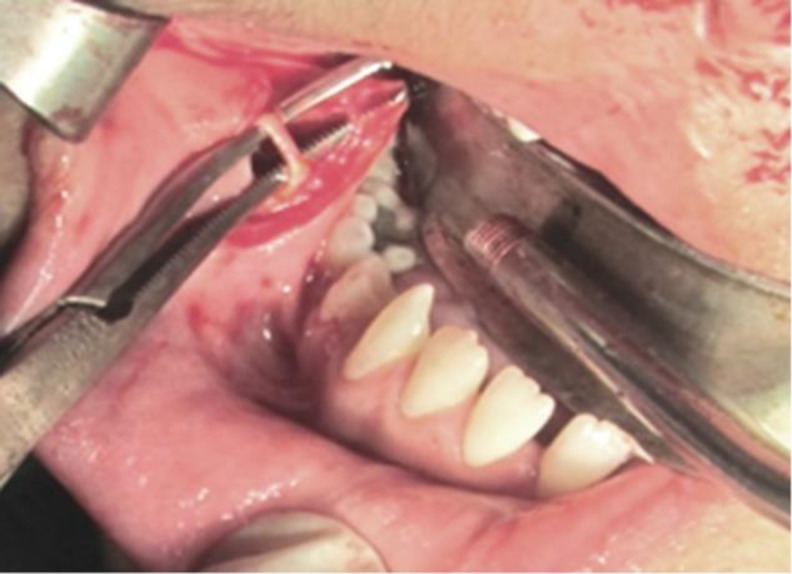
Facial artery is skletonized in the mandibular buccal vestibule

Facial vessels in the inferior border of the flap were detected with Doppler ultrasound and included in the flap design (Figure 2).

**Fig 3:**
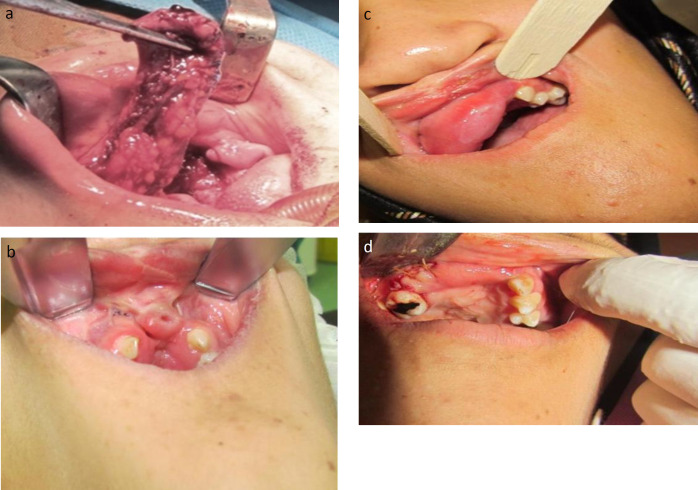
Coverage of the bone graft with FAMM flap as an interpolated flap, a. FAMM flap composed of buccal mucosa, submucosa, small part of buccinator and orbicularis muscles. b. Edentulous atrophic premaxilla with a retained decayed root. c: Interpolated flap before pedicle division(first case); d: The second case just after pedicle division

Finally, the facial artery was ligated and sharply incised at the mandibular vestibule. The flap is composed of buccal mucosa, submucosa, a small part of buccinators, and orbicularis oris muscles. The flap was turned over the bone graft and sutured to the adjacent mucosa. It was used as an interpolated flap. Three weeks later the flap pedicle was divided under local anesthesia (Figure 3).

## Results

The information of 10 patients that superiorly-based buccinator myomucosal flaps were used for the management of difficult cleft cases is displayed in Table 1.

**Table 1:** Demographic information of the patients that buccinator based myomucosal flaps are used for management of difficult cleft cases

	**Age (years)**	**Gender**	**Condition**	**Type of CLP**	**Method of flap transfer**	**Follow up (Years)**	**Hospital stay(Day)**
1	14	F	Edentulous atrophic premaxilla	BCLP	Alveolar gap	2	1
2	22	F	Edentulous atrophic premaxilla	BCLP	Alveolar gap	2	1
3	28	F	Premaxillectomy	BCLP	Alveolar gap	1	1
4	14	M	Absent premaxilla in facial cleft patient	Tessier cleft	Alveolar gap	1	1
5	14	M	Wide unilateral alveolar cleft	UCLP	Alveolar gap	1	1
6	18	F	Large palatal fistula behind premaxilla	BCLP	Transmaxillary transfer	2	1
7	20	M	Large palatal fistula	BCLP	Behind dentition	3	1
8	25	M	Wide alveolar cleft in edentulous maxilla	UCLP	Alveolar gap	2	1
9	14	M	Wide unilateral alveolar cleft with missing of three permanent tooth	UCLP	Alveolar gap	1	1
10	18	M	Premaxillectomy	BCLP	Alveolar gap	1	1

FAMM flap: Facial Artery Myomucosal flap, BCLP: Bilateral cleft lip /Palate, UCLP: Unilateral cleft lip /Palate, CLP: Cleft lip/palate

**Fig 4:**
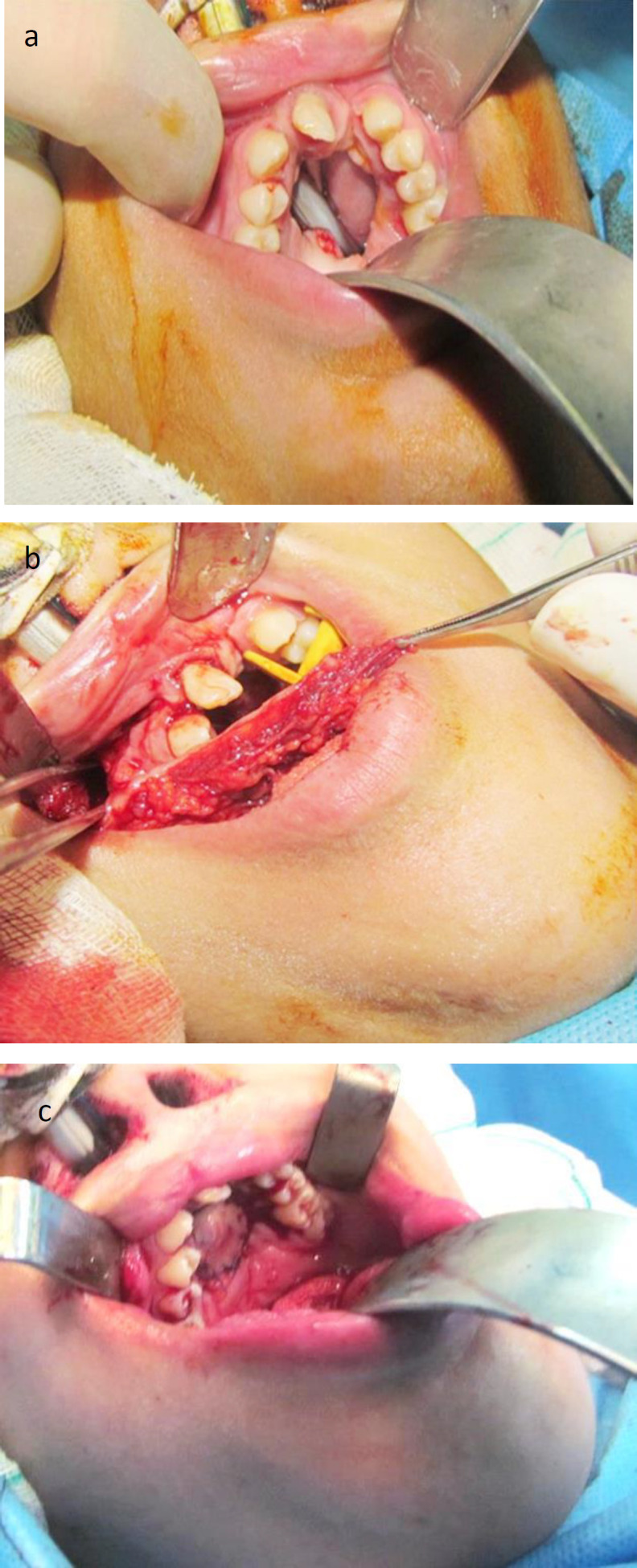
a. large palatal fistula behind premaxilla, b. Bony window is between two beaks of college pliers for transmaxillary transfer of FAMM flap, c. Immediate post-operative photograph

The majority of patients were male (6/10), and the age range of patients was obtained at 14-25 years. In one patient, this flap was transmaxillary transferred through the bony window created into the maxillary bony wall to reach the palatal fistula, while the maxillary dental arch was without the space (Figure 4).

In three patients, the inferior turbinate flap was used for nasal side coverage, while in other patients, hinge flaps were effective for nasal floor reconstruction. 

**Fig 5:**
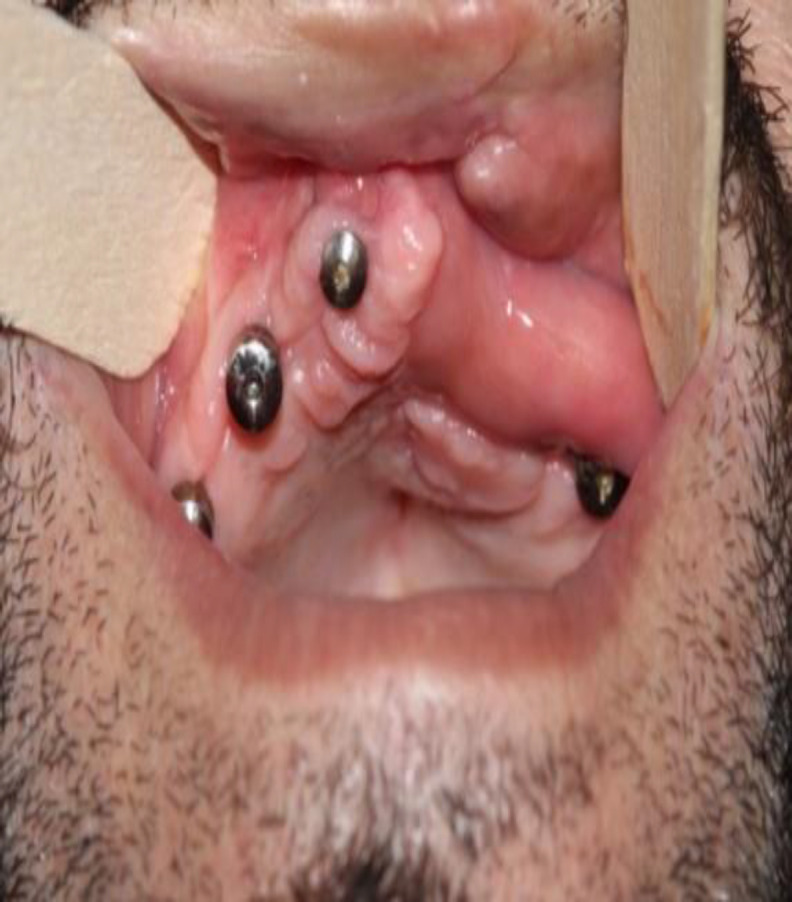
Need for flap pedicle division and local vestibuloplasty

Flap pedicle division was needed when FAMM flap is used for alveolar cleft reconstruction; nonetheless, this surgery can be performed without the need for general anesthesia (Figure 5). The length of hospital stay was one day for all patients.

## Discussion

Cleft patients with large palatal fistula are difficult cases for treatment (9). The limitation of the tongue flap in this region is the small width of the transferred flap (10). An anteriorly-based tongue flap is recommended for anterior fistula, while a posterior-based variant is better tolerated by the patient. Both of them have a blood supply of random patterns (11,12). 

Three-week attachment of the tongue to the palate for revascularization and the need for second surgery are the major drawbacks. The width of the tongue flap should be 20% more than the fistula diameter (13).

A negative factors in choosing temporal and temporoparietal fascia flaps is donor site morbidity. Injury to a temporal branch of the facial nerve and a noticeable scar in the incision line of the scalp are probable. 

Temporal flap harvest needs reconstruction options to prevent hallow deformity. Dividing this flap into anterior and posterior parts, transmaxillary transfer of anterior part to the oral cavity for fistula closure, and mobilization of the posterior part to prevent soft tissue deformity in donor site are recommended (14,15). 

The submental flap also has a linear scar below the mandible in the neck and poses a minor risk of injury to the marginal mandibular branch of the facial nerve. The scar is hidden in the shadow of the mandible but may be noticeable if a hypertrophic scar is formed (16). Reverse flow flap is more suitable than orthograde variant for palatal reconstruction, although it is mostly used in pathologic resections. Free radial forearm flap is reliable but needs training in microvascular surgery (17). Buccinator-based myomucosal flaps have the advantage of transferring oral mucosa for reconstruction (18). 

**Fig 6:**
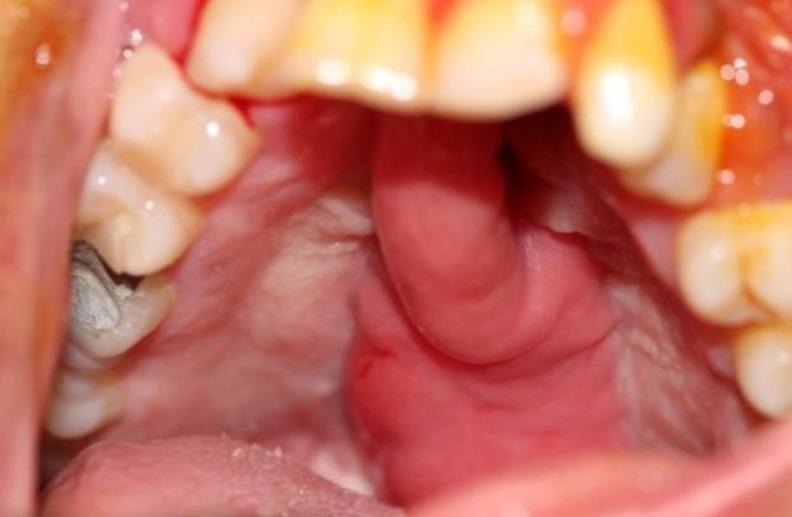
Waltzing of FAMM flap to solve the problem of recurrent palatal fistula

Mucosa is better than skin for oral cavity lining (19). Intraoral incisions, low morbidity, and vicinity to the palate are among other advantages. Posteriorly-based buccinator myomucosal flap (Bozola flap) is a neurovascular flap which contains the buccal artery and nerve; therefore, the return of sensation is anticipated (20). Other benefits include the thin nature of the flap and saliva secretion. This flap is indicated in primary cleft palate surgery (wide clefts) and as a supplementary way to increase velopharyngeal insufficiency. It has unique applications for the closure of the fistula in the junction of the hard and soft palate (21-23). Readvancement or waltzing of the FAMM flap is another capacity of this flap (Figure 6) (24,25).

The FAMM flap is an arterialized flap which can be harvested both superiorly or inferiorly (26,27). Inferiorly-based FAMM flap is supplied by facial vessels, while superiorly based FAMM flap is nourished by angular artery (28,29). 

This flap which was introduced by Pribaz in 1992 (30) is a good choice for palatal fistula closure when the palatal fistula is anteriorly located and is continuous with the maxillary alveolar cleft. The superiorly-based variant can cover the fistula up to 2.5cm. A gap in the maxillary arch to pass the flap pedicle is necessary and needs the second surgery for pedicle division (20). 

In contrast to the tongue flap, the waiting time for flap insertion and pedicle division is not frustrating for the patient (26,31).

The facial artery in the flap guarantees its viability. The paddle shape is determined by the course of facial vessels. Deep dissection is needed for including facial artery in flap thickness. 

**Fig 7:**
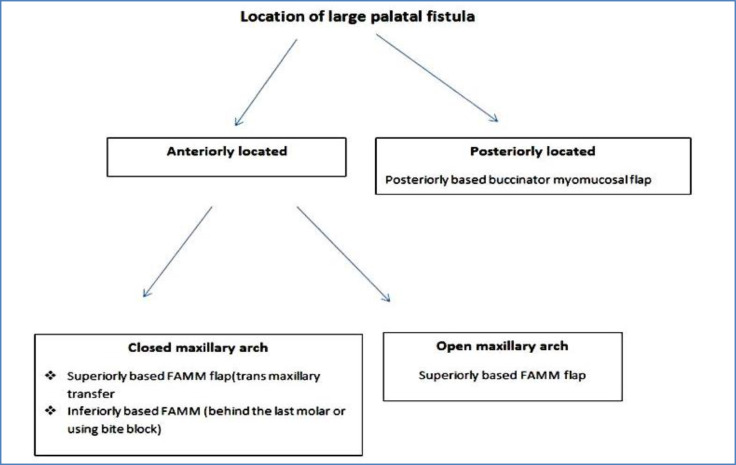
Decision tree for selecting buccinator based myomucosal flaps in management of difficult fistula

An extra length flap is needed when transmaxillary transfer of superiorly based buccinator myomucosal pedicled flap is considered. This extra length can be achieved by caudal extension of flap margins (32); nonetheless, it is not feasible in some patients with limited available mucosa. An algorithm for the management of large palatal fistula with buccinator-based myomucosal flaps is suggested in Figure 7.

## Conclusion

A posteriorly-based buccinators myomucosal flap is a good choice for the closure of a huge posteriorly located Oronasal fistula (ONF) in adult patients with cleft palate. A superiorly-based FAMM flap is a good option when the fistula is continuous with an alveolar cleft. In closed maxillary arches, transmaxillary transfer of superiorly-based FAMM flap can be considered.
